# Immunolocalization of Adrenoceptors in the Reproductive Tract of Male Domestic Cats in Comparison to Rats

**DOI:** 10.3390/ani11041049

**Published:** 2021-04-08

**Authors:** Sylwia Prochowska, Stanisław Dzimira, Alicja Tomaszek, Wojciech Niżański

**Affiliations:** 1 Department of Reproduction and Clinic of Farm Animals, Wrocław University of Environmental and Life Sciences, pl. Grunwaldzki 49, 50-366 Wrocław, Poland; wojciech.nizanski@upwr.edu.pl; 2 Department of Pathology, Wrocław University of Environmental and Life Sciences, ul. Norwida 31, 50-375 Wrocław, Poland; stanislaw.dzimira@upwr.edu.pl (S.D.); alicja.tomaszek@upwr.edu.pl (A.T.)

**Keywords:** domestic cat, rat, adrenergic receptors, epididymis, vas deferens, immunohistochemistry

## Abstract

**Simple Summary:**

In cats, semen is collected by pharmacological stimulation. The administration of a drug that stimulates α2-adrenoceptors causes the expulsion of spermatozoa into the urethra. However, as the results are not always satisfactory, this method needs to be improved. There are nine subtypes of adrenoceptors that are involved in the contraction of smooth muscle, including those in the reproductive tract, so adrenoceptors other than the α2-subtype are potential targets in any new, optimized protocol. The aim of this study was to analyze the immunolocalization of the adrenergic receptors in the reproductive tract of the male cat for the first time in this species. The expression of all adrenoceptor subtypes was noted in the peritubular smooth muscle in cats, indicating a potential clinical application for agonists of these receptors for the optimization of the pharmacological semen collection in felids. In a broader context, the development of a new procedure for semen collection in the male cat, using active substances from groups other than those currently used, will support the wider application of reproductive biotechnologies in felids.

**Abstract:**

Adrenoceptors mediate the action of the sympathetic nervous system, including the contraction of the epididymis and vas deferens. The aim of this study was to immunolocalize the adrenergic receptors in the reproductive tract of the male cat, as this information is not yet available. The epididymis and vas deferens of domestic cats and rats (the biological controls) were analyzed by immunohistochemistry to determine the localization of the α1A-, α1B-, α1D-, α2A-, α2B-, α2C-, β1-, β2-, and β3-adrenoceptors. All the receptors were expressed in the peritubular smooth muscles of the cat, but the α1D-, α2C-, and β1-adrenoceptors were not detected in this tissue in the rat. For the α2A-adrenoceptor, the intensity of immunostaining differed significantly between the caput epididymis (weakest staining) and the vas deferens (strongest staining). The presence of all the types of the receptors was also detected in the cytoplasm of the epithelial cells in all the regions of the reproductive tract. The strong expression of the α2A-adrenoreceptor suggests it has a leading role in the contraction of the reproductive tract in the cat. The presence of other adrenergic receptors in the smooth muscle of the epididymis and vas deferens indicates a potential clinical application for α1-mimetics in the optimization of pharmacological semen collection in felids.

## 1. Introduction

Adrenoceptors (adrenergic receptors) are targets for norepinephrine and epinephrine and play a crucial role in the central and peripheral actions of these catecholamines, which are mediators of the sympathetic nervous system. Three families of adrenergic receptors have been described (α1-, α2-, and β-adrenoceptors), with each family divided into three subtypes, meaning that nine distinct adrenoceptors are recognized: α1A, α1B, α1D, α2A, α2B, α2C, β1, β2, and β3 [1]. Each subtype has its own characteristics (e.g., a different tissue distribution and function (reviewed by [2,3,4])), and in some cases, the activation of the different subtypes exerts opposite effects on the organism (e.g., the activation of the α2A-adrenoceptors decreases blood pressure, whereas the activation of the α2B-adrenoceptors increases blood pressure [3]). Although adrenoceptors are recognized most commonly for their regulation of cardiac action, their constriction and relaxation of smooth muscle (e.g., vasoconstriction, bronchodilatation), and their effect on the central nervous system (e.g., the sedative action of the α2-adrenoceptors), they are widely distributed throughout the body and mediate various physiological functions, including, e.g., lipid metabolism [3,4] or the regulation of body temperature [3].

Adrenoceptors are also present in the male reproductive system, where they are involved, inter alia, in the contractility of the epididymis and vas deferens [5,6]. Alpha1-adrenoceptors seem to play a pivotal role in this process, as α1-adrenoceptor triple-knockout male mice were infertile because of an impairment in their sperm transit [7]. Moreover, men treated with α1-adrenoceptor antagonists observed ejaculatory dysfunction as a side effect [8]. Multiple pharmacological studies have been performed in various species, e.g., in rats [9,10], mice [11], rabbits [6] and guinea pigs [12,13], to elucidate the role of adrenoceptors in epididymal and/or vas deferens’ contractility. The results of those experiments showed that the α1- and α-2-agonists and β-antagonists induced/enhanced epididymal contraction, whereas the α1- and α2- antagonists blocked/decreased contractility. In addition to their insights into the molecular mechanisms of sperm transport and ejaculation, these studies laid the foundation for the manipulation of sperm transit by pharmacological agents.

In cats, the method of choice for semen collection is urethral catheterization after the administration of medetomidine, an α2-agonist [14]. The selection of medetomidine for this purpose was not based on either molecular or contractility studies, as the presence, distribution, and functionality of adrenergic receptors in the feline reproductive system have not yet been evaluated. As this pharmacological method of semen collection in cats yields variable results (particularly in terms of the large differences in the total sperm number between males [15]), it needs to be further improved. The optimization could include the administration of drugs from other groups (α1-agonists, β-antagonists) alone or in combination, but such studies should be preceded by a deeper understanding of epididymal physiology at the molecular level.

The aim of this study was to analyze the immunolocalization of the different subtypes of adrenergic receptors in the reproductive tract of the male cat.

## 2. Materials and Methods

### 2.1. Tissues

Feline epididymides with vasa deferentia were obtained from male cats (*n* = 6), aged 6 months to 2 years, intended for routine castration procedures at the Ambulatory of the Department of Reproduction and Clinic of Farm Animals, Wroclaw University of Environmental and Life Sciences. Rat epididymides with vasa deferentia were obtained from 24-week-old Wistar rats (*n* = 6), euthanized by the dislocation of the cervical vertebrae in deep anesthesia at the end of another experiment (males from the negative control group). All procedures were performed with the agreement of the Second Local Ethical Committee in Wroclaw, decision no. 24/2020. The material was fixed promptly after excision, in 7% buffered formalin for 1–5 days, and then the samples were cut into four parts (the caput epididymis, corpus epididymis, cauda epididymis, and vas deferens) and embedded in paraffin separately.

### 2.2. Antibodies

Antibodies against all nine subtypes of adrenoceptors (an anti-α1A-, anti-α1B-, anti-α1D-, anti-α2A-, anti-α2B-, anti-α2C-, anti-β1-, anti-β2-, and anti-β3-adrenergic receptor antibody, Adrenergic Receptor Antibody Explorer Kit Cat#: AK-101) were purchased from Alomone Labs (Jerusalem, Israel). All antibodies were affinity purified rabbit polyclonal IgG, designed to recognize human, rat, and mouse adrenoceptors. There are no antibodies specific for the domestic cat available on the market; however, taking into consideration the high homology (90%) of adrenoceptors between species [16], the affinity to feline receptors was expected and checked before the main experiment.

### 2.3. Immunohistochemistry

The formalin-fixed, paraffin-embedded tissues were freshly cut into 3 µm-thick sections and mounted on Superfrost Plus slides (Menzel Gläser, Braunschweig, Germany). At least ten sections from each region investigated for each individual were prepared (one per each investigated receptor plus a reserve). The sections were then dewaxed with xylene and gradually hydrated in alcohol. The sections were then boiled for 30 min in Dako Target Retrieval Solution, pH 6 (Dako, Glostrup, Denmark). The activity of the endogenous peroxidase was blocked by Peroxide Block (NovolinkTM Max Polymer Detection System, Leica Biosystems, Newcastle upon Tyne, UK). In order to measure the levels of the antigens studied, the antibodies were diluted in the IHC Diluent (Leica Biosystems, Newcastle upon Tyne, UK) to 1:100 (the anti-α1A-, anti-α1B-, anti-α1D-, anti-α2B-, anti-β2-, and anti-β3-adrenergic receptor antibody), 1:200 (the anti-α2C-adrenergic receptor antibody), 1:250 (the anti-α2A-adrenergic receptor antibody), or 1:400 (the anti-β1-adrenergic receptor antibody) according to the manufacturer’s instructions and were applied for one hour at room temperature. Next, the samples were incubated with Post Primary NovolinkTM Polymer (NovolinkTM Max Polymer Detection System, Leica Biosystems, Newcastle upon Tyne, UK) according to the manufacturer’s instructions. The reaction used 3,3’-diaminobenzidine (DAB Chromogen) as a substrate and all the sections were counterstained with hematoxylin (NovolinkTM Max Polymer Detection System, Leica Biosystems, Newcastle upon Tyne, UK). One slide per animal per antibody was prepared. In the case of a poor staining quality, the immunochemistry procedure was repeated.

Negative control experiments were performed in the absence of the primary antibody. The surrounding blood vessels, known for the presence of all subtypes of adrenoceptors [2,17], served as an internal positive control for each section. As there is no information about adrenoceptors in the feline reproductive tract, the rat was chosen as a biological control, as it is the species in which adrenoceptors have been studied most extensively [9,10,18,19,20,21,22,23,24,25,26] and is therefore considered a model species for this type of investigation.

### 2.4. Analysis of Slides

**Figure 1:**
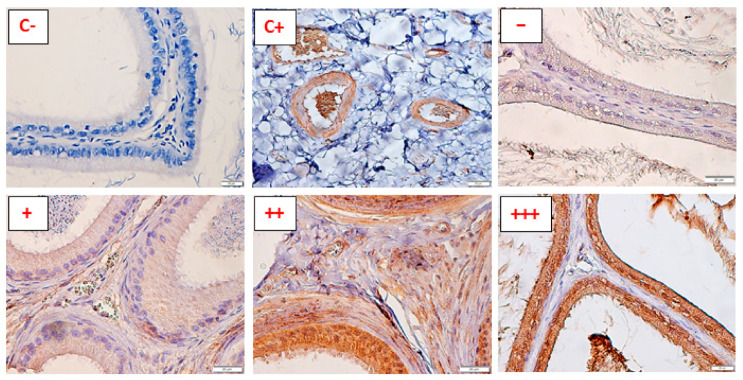
The scale of scoring the intensity of reaction—representative pictures (counterstained with hematoxylin). The white bar on the bottom right of each image represents 20 µm. (C-) = the negative control (rat caput epididymis, magnification 400×); (C+) = the positive control, average staining of the blood vessels (cat caput epididymis, magnification 400×); (−) = no positive reaction detected (rat caput epididymis, magnification 400×); (+) weak positive reaction (cat corpus epididymis, magnification 400×); (++) moderate positive reaction (cat cauda epididymis, magnification 400×); (+++) strong positive reaction (This was only seen in the epithelium. None of the smooth muscle sections were assessed as having a strong reaction) (rat caput epididymis, magnification 400×).

The sections were viewed with an Olympus BX53 microscope coupled with a UP-90 camera (Olympus Polska, Warsaw, Poland), for both the semiquantitative determination of the receptor expression (the intensity of staining scored from “−“, meaning no reaction, to “+++”, meaning a strong reaction, Figure 1), and the localization of the receptors in the smooth muscle and the epithelium of the epididymis and vas deferens. First, the slides were examined under 200× magnification for the uniformity of staining, and then the scoring of the reaction was performed under 400× magnification. At least 5 viewing fields were examined for each slide. The assessment of the slides was made independently by two researchers who were blinded to the sample’s identity. In the case of any incongruity between the assessments, the average result was noted. Different parts of the reproductive tract (the caput epididymis, corpus epididymis, cauda epididymis, and vas deferens) were analyzed separately.

### 2.5. Statistical Analysis

For the statistical analysis, the descriptive grading scale was transformed to a numerical one: 0 (−); 1 (+); 2 (++); 3 (+++). A statistical analysis was performed using the Kruskal–Wallis ANOVA (for a comparison of the reaction intensity between the different reproductive tract segments), the U-Mann Whitney test (for a comparison between the species), or the Wilcoxon signed-rank test (for a comparison of the epithelium and smooth muscle in the same section). The level of significance was set at *p* < 0.05. An elaboration of the tests was carried out using the Statistica software for Windows v.13 (StatSoft Polska Sp. z o.o., Krakow, Poland).

## 3. Results

The presence of all the receptors investigated was confirmed in the interstitial blood vessels in both species (Figure 1, C+). If not stated otherwise, the observations for each tissue/structure correspond to the mean results from six animals.

### 3.1. Smooth Muscles

**Figure 2:**
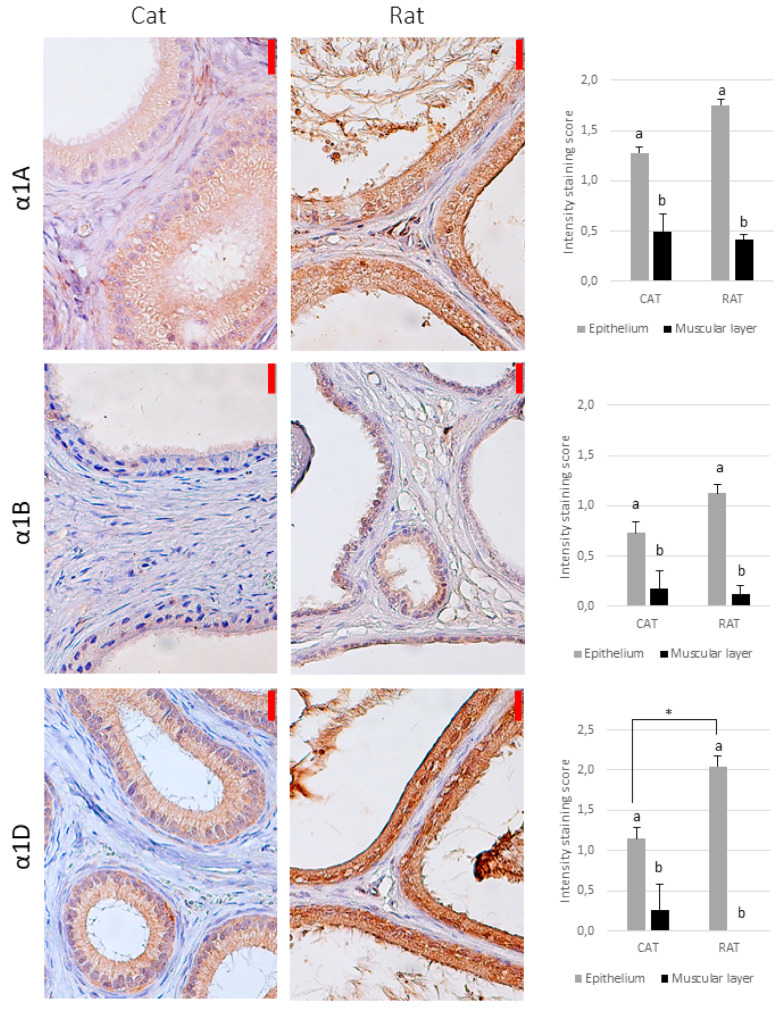
The immunohistochemical localization of the α1-adrenoceptors in the feline and rat reproductive tract − representative pictures of the epididymis (counterstained with Hematoxylin, magnification 400×). The red bar on the upper right of each image represents 20 µm. The graphs on the right present the average reaction intensity score (*n* = 6). Because of the lack of difference between the regions for most of the receptors investigated, the data are shown as the average intensity of staining for the regions analyzed and SEM, for the sake of clarity. The different letters above the bars (a, b) indicate significant (*p* < 0.05) differences between the epithelium and muscular layer within a species. An asterisk (*) indicates a significant difference between the species.

**Figure 3:**
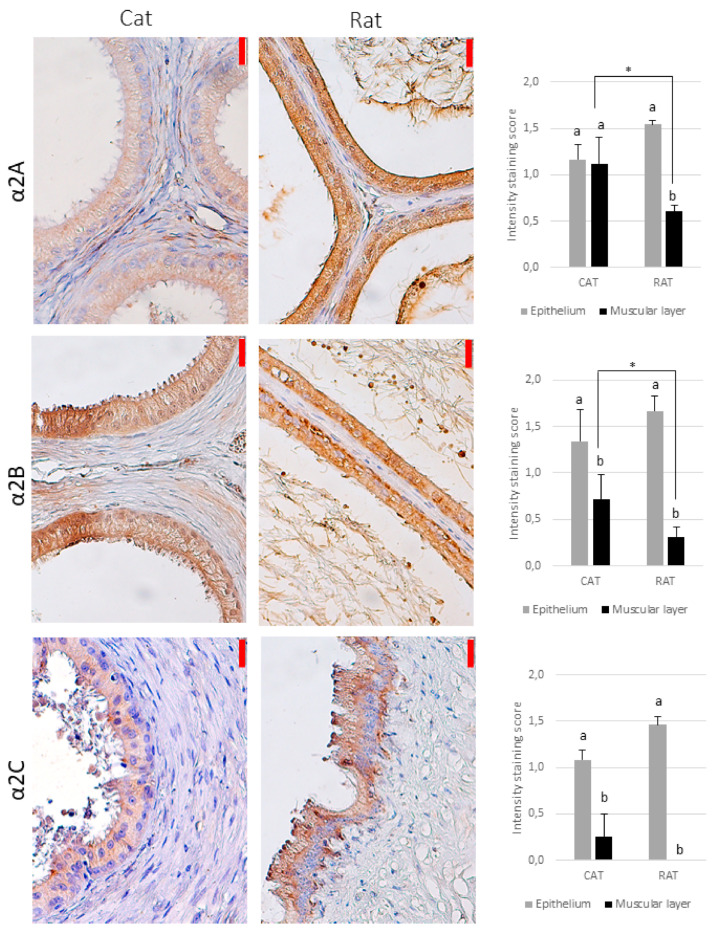
The immunohistochemical localization of the α2-adrenoceptors in the feline and rat reproductive tract **−** representative pictures of the epididymis (counterstained with hematoxylin, magnification 400×). The red bar on the upper right of each image represents 20 µm. The graphs on the right present the average reaction intensity score (*n* = 6). Because of the lack of difference between the regions for most of the receptors investigated, the data are shown as the average intensity of staining for the regions analyzed and SEM, for the sake of clarity. The different letters above the bars (a, b) indicate significant (*p* < 0.05) differences between the epithelium and muscular layer within a species. An asterisk (*) indicates a significant difference between the species.

**Figure 4:**
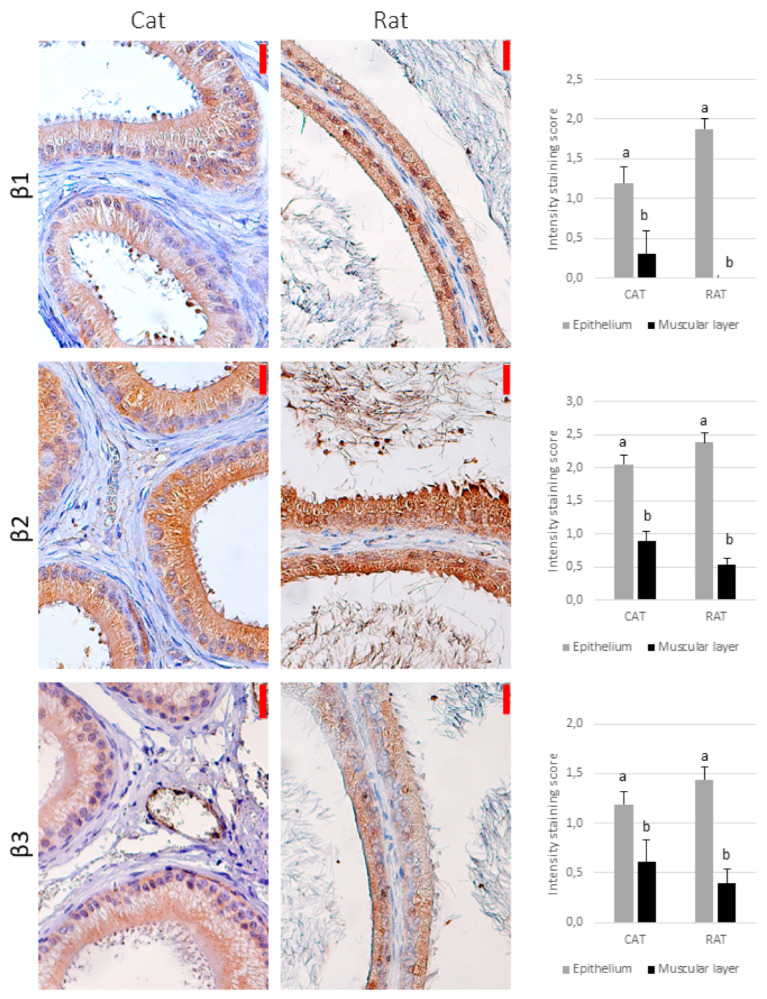
The immunohistochemical localization of the β-adrenoceptors in the feline and rat reproductive tract − representative pictures of the epididymis (counterstained with hematoxylin, magnification 400×). The red bar on the upper right of each image represents 20 µm. The graphs on the right present the average reaction intensity score (*n* = 6). Because of the lack of difference between the regions for most of the receptors investigated, the data are shown as the average intensity of staining for the regions analyzed and SEM, for the sake of clarity. The different letters above the bars (a,b) indicate significant (*p* < 0.05) differences between the epithelium and muscular layer within a species. No significant differences between the species were noted.

In the cat, there was a punctate, diffuse immunostaining of the investigated receptors in the cytoplasm for all the subtypes, although a positive signal for the α1B-adrenoceptor was observed in single individuals (regardless of the epididymal segment), and many individuals did not show a signal for the α1D-, α2C-, and β1-adrenoceptors, especially in the caput or corpus epididymis (Table 1). The strongest reaction was observed for the α2A-adrenoceptor (Table 1, Figure 2, Figure 3 and Figure 4). Only the α2A-adrenergic receptors showed significant differences between the regions, with the strongest expression in the vas deferens (Table 1).

In the rat, there was no positive reaction in the smooth muscle for the α1D-, α2C-, or β1-adrenoceptors (Figure 2, Figure 3 and Figure 4). In some individuals, the signals for the α1B- and α2B-adrenoceptors were absent from the epididymis and present only in the vas deferens. For the other adrenoceptors, the intensity of reaction was weak.

The only differences between the species were in the α2A- and α2B-adrenoceptors—the intensity of reaction was stronger in the feline vas deferens than in all the parts of the rat epididymis (caput, corpus, cauda) (Figure 3).

### 3.2. Epithelium

All the types of the investigated receptors were detected in the cytoplasm of the tubular epithelial cells in both species (in all parts of the reproductive tract), which showed a uniform, dispersed reaction (i.e., no compartmentalization) (Figure 2, Figure 3 and Figure 4). In general, the reaction was significantly stronger than in the smooth muscle, but for the α2A-adrenoceptor in the cat, the intensity of staining was similar in the epithelium and muscular layer, regardless of the epididymal region (*p* > 0.05, Figure 2).

Significant differences between the epididymal regions were noted only for the α1D-adrenoceptors in cats, with the strongest reaction in the caput epididymis and the weakest reaction in the corpus epididymis (Table 2).

There were no differences between the species except for the α1D-adrenoceptor, where the reaction intensity was stronger in the rat vas deferens than in the feline vas deferens (Figure 2). The overall expression was the strongest for the β2-adrenoreceptors, and the weakest for the α1B-adrenoreceptor (Figure 2, Figure 3 and Figure 4, Table 2).

## 4. Discussion

This is the first study to explore the immunolocalization of adrenoceptors in the reproductive tract of the domestic cat. All the subtypes were identified along the feline epididymis and vas deferens, thereby filling the knowledge gap in this field and providing useful information in terms of feline reproductive physiology and the clinical application of pharmacological methods of semen collection in this species.

The presence of the α2-adrenoceptors in the smooth muscle of the feline reproductive tract, with the strongest reaction for the α2A-adrenoceptor, suggests that this subtype is responsible for the expulsion of spermatozoa into the urethra after the administration of medetomidine [14]. However, the confirmation of the presence of other subtypes of adrenoceptors increases the possibility of using other pharmacological agents, e.g., α1-mimetics, for this purpose.

As the subject of adrenoceptors in the feline reproductive system is a blank page, in this study we performed a parallel analysis in rat, a species in which adrenoceptors have been the most extensively studied [9,10,18,19,20,21,22,23,24,25,26]. But even in rats, localization of the adrenoceptors by immunohistochemistry has only been performed for the α1-family [23], meaning that this is also the first study showing the localization of other adrenoceptor subtypes in the rat.

Our results for the α1-adrenoceptors in rats are in agreement with those of Queiróz et al. [23], who reported positive immunostaining for the α1-adrenoceptors (without division into subtypes), and with mRNA level studies [20,22] that revealed the presence of transcripts of all α1-subtypes in the rat epididymis, with a relatively low expression of the α1B-adrenoceptor. Despite the presence of mRNA for the α1D-adrenoceptor, an experiment using a radioligand binding assay failed to show the presence of this subtype in the epididymis [22] or suggested its marginal role in epididymal contractility [20]. In this study, the α1D-adrenoceptor was found intracellularly in the epithelium but not in smooth muscle, which may partially explain the previous findings.

Several studies [24,25] failed to demonstrate the presence of the α2-adrenoceptors in the rat cauda epididymis by radioligand and functional studies in vitro, despite their localization in the vas deferens [25]. These findings were questioned by Chaturapanich et al. [10], who reported a response to the α2-agonists in the cauda epididymis. In our study, the α2A- and α2B-, but not the α2C-, adrenoceptors were observed in the muscular layer of the vas deferens and (to a lesser extent) in the smooth muscle surrounding the epididymal tubules, but the intensity of reaction was very low. This weak expression may explain the inconsistency of the results obtained by the different authors.

Although pharmacological agonist–antagonist studies suggested that β-adrenoceptors are absent from the rat epididymis [19,21,24], the binding assay showed the presence of the β2-adrenoceptor in the vas deferens [9,18,26]. Our findings revealed a very low intensity of reaction in the peritubular muscular layer for the β2- and β3-adrenoceptors (mostly observed in the vas deferens) and the absence of the β1-adrenoceptors, which may explain the lack of response to the pharmacological agents in the epididymis.

The studies in other species [6,11,12,23,27] are not so numerous and often are limited to one family of adrenoceptors or to a particular segment of the reproductive tract (e.g., the cauda epididymis or vas deferens). The use of nonselective agents in many of these studies did not allow any effect to be distinguished between the α1- and α2-adrenoceptors. This makes comparisons almost impossible to perform, and thus the identification of species-specific characteristics is challenging. Despite these potential interspecies differences, we believe that the similar immunolocalization of adrenoceptors in both the species investigated in this study allows for the transfer of knowledge from the rat to the domestic cat. As studies in the rat showed the importance of the α1-adrenoceptors for the contraction of the epididymis and vas deferens, it seems reasonable to assume that this is also the case for the cat. Therefore, the inclusion of α1-agonists in the pharmacological semen collection protocol for the cat is worth further investigation.

β-adrenoceptors are linked to smooth muscle relaxation [17], so it might be anticipated that the administration of their antagonists would enhance muscle constriction in the reproductive tract. However, in rats, neither the β-adrenoceptor agonists nor the antagonists affected epididymal contractility [19,21,24], and in our study, the intensity of reaction for the β-adrenoceptors was similarly low for the cat. In light of these results, β-blockers seem to be poor candidates for the optimization of semen collection in the cat. On the other hand, a response to the β-agonists and antagonists was observed in the vas deferens of the rat [26], rabbit [6], and guinea pig [13]. As our study also showed a positive reaction in this segment of the reproductive tract, β-antagonists may still be useful for clinical application in cats, at least as potentiating agents.

In this study, the only significant difference between the regions (the caput, corpus, cauda epididymis, and vas deferens) in the cat was for the α2A-adrenoceptor, although differences have been reported for the different subtypes in the rat [22,23,25] and other species [23,27]. This lack of difference in our study could be related to the subjective assessment and the semiquantitative character of scoring the immunoreaction’s intensity. More accurate quantitative methods must be applied to examine this issue, so we are currently studying the mRNA and protein levels.

The adrenoceptors in the reproductive tract are typically studied in the context of smooth muscle contractility; however, their presence has also been confirmed in epithelial cells [23,28,29], as was also observed in our study. It has been shown that the α1-adrenoceptors in the epididymis play a role in electrolyte transport [28] and protein processing [29]. The presence of functional intracellular α1-adrenoceptors has been described in the literature [23,30,31], and it has become evident that the distribution and function of adrenergic receptors are much more complex and diverse than previously considered [17]. The domestic cat is a neglected species in this field, and, based on the wide use of adrenergic receptor agonists and antagonists in clinical practice, further studies should be performed to extend our knowledge and allow these drugs to be used to their full potential while avoiding side effects, including those that impair fertility.

## 5. Conclusions

The strong α2A-adrenoceptor expression in the muscular layer of the feline reproductive tract suggests that this is the main adrenoceptor involved in epididymal and vas deferens’ contractility in the domestic cat, and confirms the mechanism of semen collection by urethral catheterization after the administration of α2-mimetics. The presence of other adrenergic receptors in the smooth muscle of the epididymis and vas deferens indicates a potential clinical application of α1-mimetics for the optimization of pharmacological semen collection in felids.

## Figures and Tables

**Table 1:** The semiquantitative assessment of the intensity of reaction in the muscular layer of the epididymal tubuli/vas deferens in domestic cats. The data are presented as the median score of the intensity of immunostaining (*n* = 6).

Adrenoceptor Subtype		Epididymis		Vas Deferens
	*Caput*	*Corpus*	*Cauda*	
α1A	−/+	−/+	+	−/+
α1B	−	−	−	−
α1D	−	−	−	−/+
α2A	− ^a^	+ ^a^	+/++ ^a,b^	++ ^b^
α2B	−/+	−/+	+	+
α2C	−	−	−/+	+
β1	−	−	−/+	+
β2	−/+	+	+	+
β3	−/+	+	−/+	+

− no positive reaction detected; + weak positive reaction; ++ moderate positive reaction; +++ strong positive reaction. The different letters (^a,b^) indicate significant differences between the reproductive tract segments (*p* < 0.05).

**Table 2:** The semiquantitative assessment of the intensity of reaction in the epithelium of epididymal tubuli/vas deferens in domestic cats. The data are presented as the median score of the intensity of immunostaining (*n* = 6).

Adrenoceptor Subtype		Epididymis		Vas Deferens
	*Caput*	*Corpus*	*Cauda*	
α1A	+	+/++	+	+
α1B	+	+	**−**/+	−/+
α1D	++ ^a^	−/+ ^b^	+ ^a,b^	+ ^a,b^
α2A	+	+	++	+
α2B	+	+	+/++	+
α2C	+	+	+	+/++
β1	+	+/++	+/++	+
β2	++	++	++/+++	++
β3	+	+	+	+/++

− no positive reaction detected; + weak positive reaction; ++ moderate positive reaction; +++ strong positive reaction. The different letters (^a,b^) indicate significant differences between the reproductive tract segments (*p* < 0.05).

## Data Availability

The data that support the findings of this study are available from the corresponding author [S.P.], upon reasonable request.
